# Book Notices

**Published:** 1880-11-20

**Authors:** 


### *BOOK NOTICES.*

DISEASES OF THE THROAT AND NOSE. By Morrell Mackenzie, M. D., London, Senior Physician to the Hospital for Diseases of the Throat and Chest; Lecturer on Diseases of the Throat at the London Hospital Medical College, etc. Vol. 1, Diseases of the Pharynx, Larynx and Trachea. 8vo, pp. 600 Price $4.00. Philadelphia: Pressley & Blakiston.

The Dublin Journal thus remarks of this work: "We have long felt the want of a thoroughly practical and systematic treatise on diseases of the throat and nasal passages. Admirable passages have from time to time appeared, which have dealt with the various affections occurring in the naso-pharyngeal and laryngeal organs, but up to the present no standard work has been written. Any one familiar with laryngoscopic work must at once appreciate the valuable addition now made to this special department in the work before us. The entire work, of which only the first volume has as yet appeared, will include the consideration of affections of the pharynx, larynx, trachea, œsophagus, nasal cavities and neck. The matter now presented complete for the first time is the result of the author s large and unrivaled experience, both in hospital and private practice, extending over a period of twenty years. His constant labor in this special field, and the accurate methods adopted at the Throat Hospital for recording every feature of moment in the history and treatment of the cases there attending—the systematic registration of which must have struck any visitor—give to this final compilation of Dr. Mackenzie a tenfold value. There can be but one verdict of the profession on this manual—it stands without any competitor in medical literature, as a standard work on the organs it professes to treat of.

A PRACTICAL TREATISE ON NASAL CATARRH. By Beverly Robinson, A.M., M.D., Paris, Lecturer upon Clinical Medicine at the Bellevue Hospital Medical College, New York; Physician to St. Luke's and Charity Hospital, etc. New York: William Wood & Co., 27 Great Jones street, 1880.

The author, at the close of the preface, thus remarks: "I have wished especially to write a succinct, thorough, compact account of personal experience and convictions, and thus, if possible, render it valuable as a practical guide to others" No works are so valuable as those drawn from practical observation and experience. Here we have one of the opprobria of the profession dealt with by a learned, observing and practical man. The work is illustrated, neatly gotten up, and contains 176 pages of valuable information.

TREATISE ON THERAPEUTICS. Translated by D. F. Leonard, M.D., from French of A. Trousseau, Professor of Therapeutics in the Faculty of Medicine of Paris; Physician to the Hotel Dieu, etc.; and H. Pedoux, Member of the Academy of Medicine; Honorary Physician to the Hospitals; Honorary President of the Soc. de Thereapeutique; Honorary Member of the Royal Legion, etc. Ninth edition; revised and enlarged, with the assistance of Constantine Paul, Professor Agrege in the Faculty of Medicine of Paris; Physician to the Hospital St. Antoine, etc. Vol. III. New York: William Wood & Co., 27 Great Jones street, 1880.

This is certainly a valuable work. Thirty-eight pages are devoted to facts and information on *Anaesthetics*, fifty-eight pages to *Anti-Spasmodics*, twenty-two pages to *Neurosthenic Tonics*, more than one hundred pages to Excitants. thirty-two pages to *Sedatives* and Contro-Stimulants, and twelve pages to Anthelmintics. The work is exceedingly useful, containing much new, interesting and practical information.

ACTS OF THE LOUISIANA LEGISLATURE, ESTABLISHING AND REGULATING QUARANTINE FOR THE PROTECTION OF THE STATE, ETC.; RULES AND REGULATIONS OF THE BOARD OF HEALTH OF THE STATE OF LOUISIANA, AND HEALTH ORDINANCES OF THE CITY OF NEW ORLEANS, ETC. By Joseph Jones, M.D., President of the Board of Health of Louisiana. New Orleans: J. S. Rivers, Stationer and Printer, 1880.

In these times of interest and controversy on Sanitary Science, this work is timely, and will be in demand by the profession and the public.

THE NEW CYCLOPEDIA OF DOMESTIC ECONOMY AND PRACTICAL HOUSEKEEPER, adapted to all classes of society, and comprising subjects connected with the interests of every Family, such as Domestic Education, Houses, Furniture, Duties of Domest cs, the Store-Room, Marketing, Table and Attendance, Care and Training of Children, Care of the Sick, Preparation of Food for Children and Invalids, Preservation of Health, Domestic Medicine, the Art of Cooking, Perfumery, the Toilet, Cosmetics, etc. Five thousand Practical Receipts, and Maxims from the English, French, German, etc. Illustrated with 200 Engravings. By Mrs. E. F. Ellet, author of "The Women of the Revolution." Norwich, Conn.: Henry Bill Pub. Co. Ira A. Smith, Milford, Mass., General Agent.

WALSH'S PHYSICIANS' COMBINED CALL BOOK AND TABLET. Fifth edition. Published by Ralph Walsh, M.D., Washington, D. C. Mailed, pre-paid, upon receipt of $1.50, and for sale by all booksellers. It contains a Calendar Table of Doses, Abbreviations, Poisons and Antidotes, Hypodermic Formulae, Tests for Urine, Disinfectants, Obstetric Table, Incompatibles, Weights, etc. A very handy memorandum and exceedingly useful to the practitioner.

A SYSTEM OF MEICINE: Edited by J. Ruscell Reynolds, M. D., T R. S., Fellow of the Royal College of Physicians of London, etc., with numerous additions and illustrations. By Henry Hartshorn, A. M., M. D.,, Fellow of the College of Philadelphia, etc., in three volumes. Henry C. Lea & Son, Philadelphia.

We have noticed the other volumes of this standard work on Practice, and now take pleasure in commending the third and last volume, now before us. The volume treats of diseases of Dijestive, Blood-glandular, Urinary, Reproductive and Cutaneoue Systems. As a work of reference, Reynold's System of Medicine is the most thorough and complete in the English language. Each subject has assigned it a distinct author, whose special merits are fully and clearly put before the reader. We know of no work whose value so appeals to the wants of the profession, and so important to the practitioner.

